# The diversity of speech-perception difficulties among autistic individuals

**DOI:** 10.1177/23969415241227074

**Published:** 2024-01-27

**Authors:** George J Bendo, Alexandra Sturrock, Graham Hanks, Christopher J Plack, Emma Gowen, Hannah Guest

**Affiliations:** UK ALMA Regional Centre Node, Jodrell Bank Centre for Astrophysics, Department of Physics and Astronomy, 5292 The University of Manchester, Manchester, UK; Division of Human Communication, Development and Hearing, School of Health Sciences, 5292 The University of Manchester, Manchester, UK; Department of Psychology, 7315 The University of Sheffield, Sheffield, UK; Manchester Centre for Audiology and Deafness, School of Health Sciences, 5292 The University of Manchester, Manchester, UK; Division of Neuroscience and Experimental Psychology, School of Biological Sciences, 5292 The University of Manchester, Manchester, UK; Manchester Centre for Audiology and Deafness, School of Health Sciences, 5292 The University of Manchester, Manchester, UK

**Keywords:** Autism spectrum disorder, auditory processing, comprehension, hearing impairment, quality of life

## Abstract

**Background & aims:**

Communicative and sensory differences are core autistic traits, yet speech-perception abilities and difficulties among autistic individuals remain poorly understood. Laboratory studies have produced mixed and inconclusive results, in part because of the lack of input from autistic individuals in defining the hypotheses and shaping the methods used in this field of research. Little in-depth qualitative research on autistic experiences of speech perception has been published, yet such research could form the basis for better laboratory research, for improved understanding of autistic experiences, and for the development of interventions. Existing qualitative research describes widespread autistic listening differences with significant impacts, but these results rely on data gathered via oral interviews in a small sample. The present study addresses these limitations and employs a mixed-methods approach to explore autistic listening experiences.

**Methods:**

We gathered survey data from 79 autistic individuals aged 18–55 without diagnosed hearing loss. The questionnaire included 20 closed-set questions on listening abilities and difficulties and three free-text questions on listening experiences. The free-text questions underwent deductive content analysis using a framework composed of themes from previous interview data on listening experiences (including auditory differences, contributing factors, impacts, and coping strategies). Concepts in the free-text data that were not part of the analysis framework were analyzed inductively.

**Results:**

In the closed-set data, participants reported listening difficulties in most specified environments, but complex background sounds and particularly background voices caused the most difficulty. Those who reported listening difficulties expressed having substantially greater difficulties than other people the same age. Participants indicated multiple impacts from listening difficulties, most prominently in their social lives. Concepts in the free-text data strongly supported previous interview data on listening differences and factors that affect listening ability, especially the diversity of types of listening difficulties. Consistent with the closed-set data, background-sound complexity and concurrent voices were especially troubling. Some concepts in the free-text data were novel, particularly difficulties with remote, broadcast, and recorded audio, prompting the creation of new themes.

**Conclusions:**

Both forms of data indicate widespread listening differences—predominantly listening difficulties—affecting most autistic adults. Diverse types of listening difficulty are evident, potentially indicating heterogeneous underlying mechanisms, and complexity of background noise is consistently identified as an important factor. Listening difficulties are said to have substantial and varied impacts. Autistic adults are keen to share coping strategies, which are varied and usually self-devised.

**Implications:**

Based on both the quantitative and qualitative results, we provide recommendations to improve future research and support the autistic community. The data-revealing types of listening difficulties can guide better quantitative research into underlying mechanisms. Such research should take into account potential heterogeneity in listening difficulties. Suggestions for optimized collection of self-report data are also offered. Additionally, our results could be used to improve societal understanding of autistic listening differences and to create beneficial interventions for and with autistic individuals. Moreover, given the willingness of the autistic community to share coping strategies, systematic collation of these strategies could form the basis for self-help and clinical guidance.

## Introduction

Recent reports indicate that up to 2.2% of the population may be autistic (Dietz et al., 2020). Among the main diagnostic criteria for autism are difficulties with social communication and interaction (American Psychiatric Association, 2013), yet speech-perception abilities and difficulties among autistic people remain poorly understood by researchers, despite the importance of speech for human communication. While anecdotal evidence about difficulty with speech perception is available (Grandin, 1992; Attwood, 2007), laboratory studies on the topic have produced mixed and inconclusive results (Sturrock et al., 2022).

We argued previously that an important shortcoming of such research may be the absence of autistic voices in shaping its hypotheses and methods, potentially undermining its capacity to capture the aspects of speech perception that cause autistic people difficulty in real-world listening environments (Sturrock et al., 2022). Listening tasks are generally designed to test narrow aspects of listening. For example, they employ specific types of background noise to “mask” target sounds, ranging from simple “white” noise to recorded soundscapes and competing talkers. If the listening conditions used in a task are not representative of those that cause difficulty in daily life, the task will be insensitive. Moreover, the nature of speech-perception skills and difficulties may vary among autistic individuals (Sturrock et al., 2022), reducing study power and potentially obscuring listening differences if such heterogeneity is not considered. Prior studies have often been designed using hypotheses and methods uninformed by autistic insight. Researchers have tended to assume a single underlying cause of perceptual differences in autism, overlooking potential heterogeneity (Happé et al., 2006).

The need to build a foundation for better laboratory research in this area is one reason for obtaining detailed feedback from autistic individuals on their personal experiences with speech perception. However, more broadly, this information will allow us to better understand the lived experiences of the autistic community—an important branch of aural diversity (Drever & Hugill, 2022; Davies, 2023; Rosas-Pérez & Galbrun, 2023). It should inform measures to improve the communicative environment for this community, including behavior changes for communication partners, better living and working environments, and improved services for autistic individuals (Leadbitter et al., 2021). Finally, we believe that community-generated research questions should be central to autism research. The present project was established when two of our autistic co-authors (GB and GH) identified speech perception as an autism research priority.

In discussing the purpose and design of this research, it is important to acknowledge both the value and the limitations of self-report data on perceptual abilities. We cannot be certain that an autistic person (or any person) who believes they have listening difficulties relative to others will exhibit corresponding deficits in tests of listening performance. Even if one group of people reports greater difficulties than another, this may not correspond to measurable disparities. One group may hold itself to a higher standard than the other; what some people regard as “pretty much keeping up,” others might regard as “really struggling.” For this reason, the present study does not compare self-report data from autistic and nonautistic people, nor perform any inferential analysis of quantitative data; all analyses are descriptive or qualitative. We believe that high-quality self-report data do not substitute for high-quality quantitative data on listening differences; the former should provide a foundation for the latter, as well as increase understanding of individuals’ lived experiences.

While qualitative studies have explored autistic individuals’ first-hand sensory experiences (Ashburner et al., 2013; Jones et al., 2003; Kirby et al., 2015; Robertson & Simmons, 2015, 2018), they have generally lacked in-depth data on speech perception. Sturrock et al. (2022) recently analyzed interview data on autistic adults’ experiences and difficulties with speech perception, identifying six key themes and a rich array of subthemes. Interviewees described a number of different types of listening difference, as well as factors that contribute to or compound these differences. They indicated the impacts of listening difficulties on many areas of their lives, and strategies used to manage listening difficulties. However, while this interview-based approach yielded rather deep data on the topic, the sample was small, potentially under-representing heterogeneity in the autistic population. Moreover, its reliance on in-person and video-link interviews likely biased the sample away from individuals who find these communication modes difficult or unpleasant (Buckle, 2023).

In this work, we present complementary survey-based data from a larger sample. Unlike the prior interview-based study, the present work should provide viewpoints from people less comfortable with oral communication. Our survey consists of two parts: a closed-set portion analyzed quantitatively and a free-text portion addressed via deductive content analysis. Each has its own distinct value in understanding and responding to autistic individuals’ speech-perception abilities and difficulties.

## Methods

Wherever possible, our reporting of the study methods conforms to the Consolidated Criteria for Reporting Qualitative Research (COREQ) checklist; the exceptions are checklist items irrelevant to remotely collected survey data (see Supplementary Material item SM1 for the completed checklist). Since researchers’ prior experience and assumptions may influence their judgments in qualitative analysis, we will state the backgrounds of our research team. AS (PhD) is a female lecturer, autism researcher, and specialist speech and language therapist without an autism diagnosis. CP (PhD) is a professor of auditory neuroscience without an autism diagnosis. EG (PhD) is a senior lecturer without an autism diagnosis and chair of the Autism@Manchester research network. GB (PhD) is a professional astronomy researcher with an autism diagnosis who is collaborating on multiple psychology research projects. GH (BSc) is a psychology researcher with an autism diagnosis. HG (PhD) is a research fellow in auditory neuroscience. AS and EG have extensive prior experience in qualitative research. HG, GB, and GH have some prior experience in qualitative research and received training and guidance from AS and EG.

### Participants

Participants were recruited via online message boards and networks, such as Autism@Manchester, Salford Autism, Aspire, and Reddit (Shatz, 2017). Participants were required to be between 18 and 55, to self-report either having an autism diagnosis (including Asperger's syndrome) or be actively seeking a diagnosis, and to never have been diagnosed with hearing loss.

A total of 84 individuals completed the survey over the course of two months. The answers from five people were excluded: three because they had been diagnosed with hearing loss, one because they self-reported as “not autistic,” and one because they failed to answer the screening questions. This left an evaluable sample size of 79 (see Table 1 for descriptive statistics). Notable is the high proportion of female participants despite higher rates of autism diagnosis in males (Loomes et al., 2017). This is likely to reflect several factors, including a greater willingness to participate in research among young women than young men (Dunn et al., 2004), increasing recognition and diagnosis of autism in girls and women (Russell et al., 2022), and deliberate engagement by our team with female-friendly autistic communities.

**Table 1.** Statistical information about the participants.

Total evaluable responses	79
Male	22 (28%)
Female	50 (63%)
Other gender	7 (9%)
Age range	18–55 (mean: 30.6; median: 30)
Diagnosed autistic	55 (70%)
Seeking an autism diagnosis	24 (30%)

### Survey design

The survey was implemented in REDCap (Harris et al., 2009) and was composed of three screening questions on personal characteristics, 20 closed-set questions on listening abilities and difficulties, and three free-text questions on listening experiences (see Supplementary Material item SM2 for survey wording). The questions were written and refined collaboratively by AS, GB, GH, and HG, to meet the needs of autistic participants (Gowen et al., 2019).

The screening questions were intended to identify any individuals who were not autistic, who had a diagnosed hearing loss that would complicate the interpretation of the causes of their listening abilities, or who may have been affected by age-related hearing loss.

Two groups of closed-set questions asked about individuals’ experiences of listening to a person speaking in seven different auditory environments. The first group of questions asked how easy it was to hear and understand speech in: (1) a quiet environment, (2) moderate mechanical noise, (3) loud mechanical noise, (4) moderate-volume music, (5) loud music, (6) one or two other people talking, and (7) many people talking. The second set asked the participants whether they perceived each of these situations as causing them greater listening difficulty than most people their age. The seven different listening situations were chosen to reflect background noises that differed in complexity and sound level. The third set of closed-set questions asked about five potential impacts of listening difficulties: whether they stopped the participant from doing anything, affected their education or career, affected their social life, affected their feelings about themself, and affected the impression they make on other people. To avoid unduly leading the participants, the questions on listening difficulty relative to others and the impacts of listening difficulties were not posed unless the participants had previously reported relevant listening difficulties.

Two free-text questions analyzed for this report asked about any listening situations that the participant found particularly easy or difficult and anything that made listening harder or easier. In practice, the responses to these questions addressed similar topics, and the responses to the two questions were often similar, so the responses from these two free-text questions were pooled for analysis. The survey concluded with a yes/no question about whether additional research into speech perception in autistic people was felt to be a good idea (not analyzed for this report) and a free-text question seeking participants’ suggestions on research priorities.

The closed-set and free-text data were analyzed separately and sequentially in the order described here. Findings are also reported separately but integrated in the Discussion to draw out prevailing themes.

### Analysis of closed-set data

**Figure 1.**
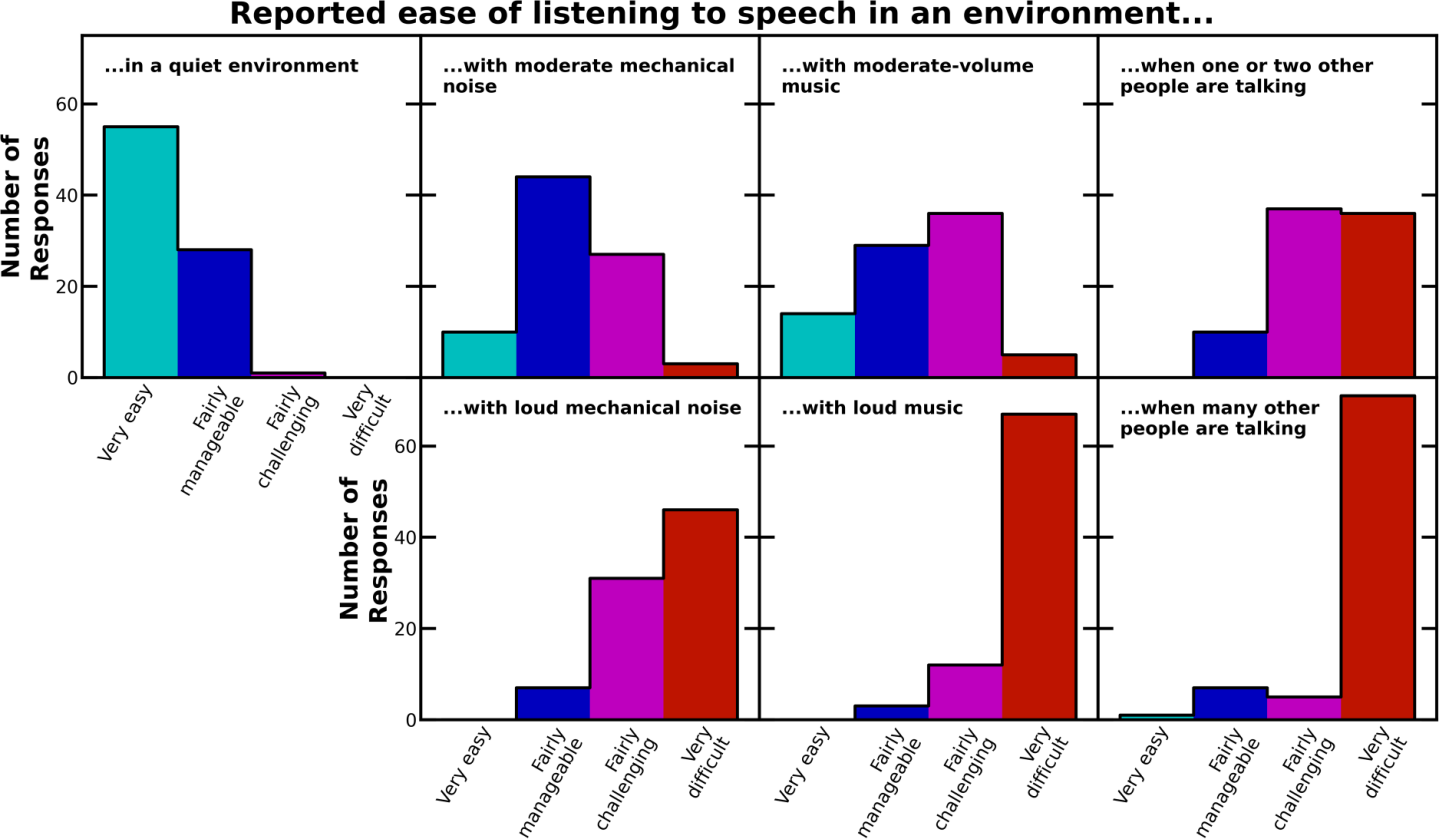
Histograms of the responses to the questions asking participants about how easily they could listen to conversations in seven different environments.

**Figure 2.**
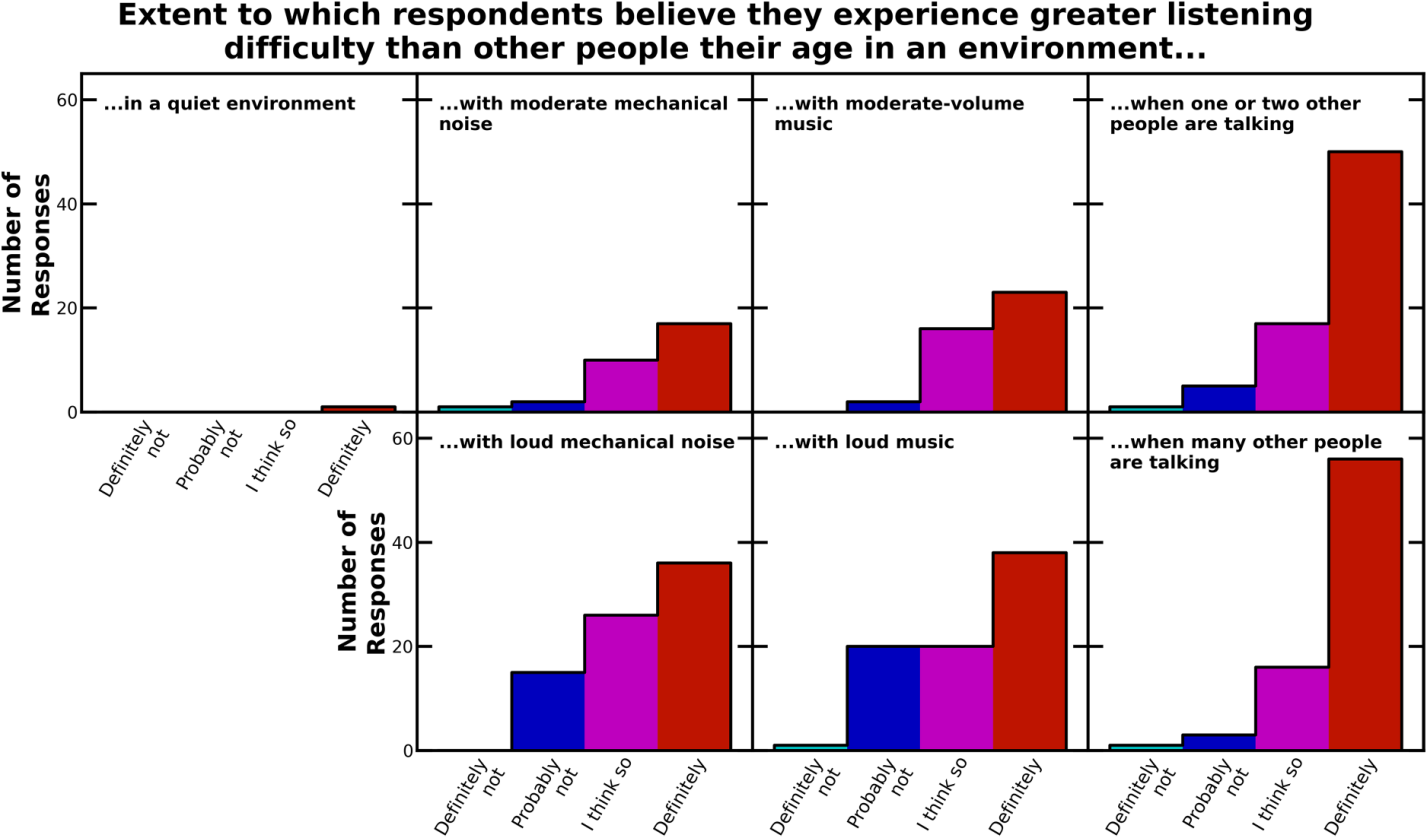
Histograms of the responses to the questions asking participants about whether they had greater difficulty than other people their age when listening to conversations in seven different environments.

**Figure 3.**
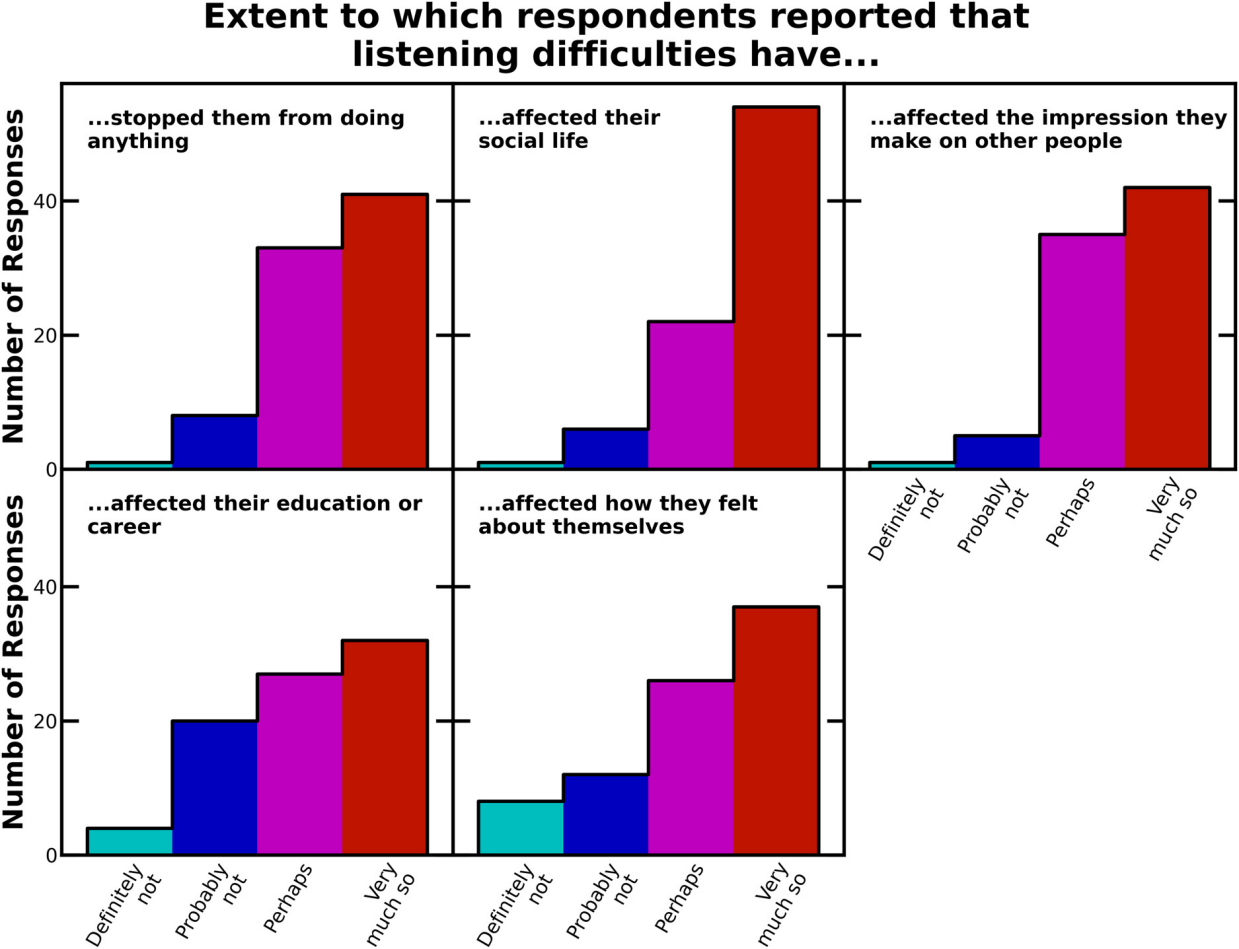
Histograms of the responses to the questions asking participants about the impact of listening difficulties on their lives.

The closed-set analyses and resulting statistics are descriptive rather than inferential. No hypotheses were defined a priori, so results should be regarded as hypothesis-generating, not hypothesis-confirming. Closed-set questions were grouped into three sets: those examining listening difficulty (*n* = 7), those examining listening difficulty relative to others the same age (*n* = 7), and those examining impacts of listening difficulty. For each question, the distributions of responses across the four options were recorded as percentages of the total responses to that question (Tables 2 to 4) and plotted as histograms (Figures 1 to 3).

**Table 2.** Responses to “Ease of listening…” questions.

Ease of listening to a person talking in an environment with…	Response			
	Very easy	Fairly manageable	Fairly challenging	Very difficult
… no background noise	52 (66%)	26 (33%)	1 (1%)	0 (0%)
… moderate mechanical noise	9 (11%)	43 (54%)	25 (32%)	2 (3%)
… loud mechanical noise	0 (0%)	6 (8%)	31 (39%)	42 (53%)
… moderate-volume music	13 (16%)	28 (35%)	34 (43%)	4 (5%)
… loud music	0 (0%)	3 (4%)	12 (15%)	63 (81%)
… one or two other people are talking	0 (0%)	9 (12%)	36 (46%)	33 (42%)
… many other people are talking	0 (0%)	7 (9%)	5 (6%)	67 (85%)

**Table 3.** Responses to “Greater difficulty…” questions.

Greater difficulty than other people of the same age when listening to a person talking…	Response				
	Definitely not	Probably not	I think so	Definitely	Total responses
… in a quiet environment	0 (0%)	0 (0%)	0 (0%)	1 (100%)	1
… with moderate mechanical noise	1 (4%)	1 (4%)	10 (37%)	15 (56%)	27
… with loud mechanical noise	0 (0%)	15 (21%)	24 (33%)	34 (47%)	73
… with moderate-volume music	0 (0%)	2 (5%)	15 (39%)	21 (55%)	38
… with loud music	1 (1%)	20 (27%)	18 (24%)	36 (48%)	75
… when one or two other people are talking	1 (1%)	5 (7%)	17 (25%)	46 (67%)	69
… when many other people are talking	1 (1%)	3 (4%)	15 (21%)	53 (74%)	72

**Table 4.** Responses to impact questions.

Have listening difficulties ever…	Response			
	Definitely not	Probably not	Perhaps	Very much so
…stopped you from doing anything?	1 (1%)	8 (10%)	31 (39%)	39 (49%)
…affected your education or career?	4 (5%)	19 (24%)	27 (34%)	29 (37%)
…affected your social life?	1 (1%)	6 (8%)	22 (28%)	50 (63%)
…affected how you feel about yourself?	7 (9%)	12 (15%)	25 (32%)	35 (44%)
…affected the impression you make on other people?	1 (1%)	5 (6%)	33 (42%)	40 (51%)

### Analysis of free-text data

Free-text data were analyzed principally via deductive content analysis, an approach that tests existing concepts against new data (Kyngäs and Kaakinen, 2020). The units of analysis were themes. The theme list of Sturrock et al. (2022; obtained from interviews on listening experiences with autistic adults) served as our analysis matrix (see Supplementary Material item SM3). This approach was supplemented, where necessary, by inductive content analysis of concepts that were evident in the survey responses but not in the analysis matrix.

It is important to note that the free-text survey questions were not designed to deliberately elicit responses relating to the themes in the analysis matrix (that is, the themes reported by Sturrock et al., 2022). Instead, broad questions on listening experiences were posed: “Are there any listening situations you find particularly easy or difficult?” and “Is there anything that makes listening harder or easier?,” along with a question about suggested priorities for auditory research in autism. Given this language, it would be reasonable to expect most responses to relate to the first three main themes of the analysis matrix only (“Auditory anomalies,” “Acoustic contributing factors,” and “Nonacoustic contributing factors”), since no questions or prompts were provided to draw out responses relating to the remaining themes (“Compounding factors,” “Impact,” and “Coping strategies”). Nonetheless, all six main themes (and their sub- and tertiary themes) were included in the matrix, and some responses relating to the latter themes were observed.

In the first stage of the analysis, researchers GB and HG independently coded all free-text responses against the matrix and recorded their codes in Excel (Supplementary Material item SM3). For each main theme, subtheme, and tertiary theme in the matrix, they recorded whether concepts consistent with the theme were present in or absent from the participant's data, along with the relevant quote(s). They also recorded any additional concepts they believed to be evident, recording the same information as for the deductive analysis.

This stage was followed by five analysis meetings. At these meetings, the researchers systematically proceeded through each cell of the matrix. They compared judgments, discussed disagreements, and ultimately entered one of the following codes for every cell: (a) agreed as present from the start, (b) agreed as absent from the start, (c) initial disagreement but ultimately agreed as present, (d) initial disagreement but ultimately agreed as absent, and (e) no agreement reached. At the fourth meeting, they inductively analyzed concepts that were supplementary to the analysis matrix, discussing whether each concept was sufficiently represented in the sample and sufficiently independent to warrant the addition of a fresh theme. Ultimately, the addition of 12 tertiary themes was agreed upon. At the fifth meeting, the researchers discussed which of these fitted under an existing main theme or subtheme, and whether the creation of any new main themes or subthemes was warranted; 11 of the 12 fitted under an existing main theme or subtheme. This left one tertiary theme that did not fit under any existing main theme or subtheme, which was left as a “Minor” tertiary theme. Final email communications were used to check judgments, correct any recording errors in the populated matrix, and perform frequency counts.

As a credibility check of the resulting themes, GB and HG shared with co-authors AS and GH a table containing the new tertiary themes and a representative quote for each (Supplementary Material item SM4). AS and GH judged the quotes as consistent with the newly emerged themes.

## Results

### Closed-set data

Table 2 lists the responses to the questions about the ease of listening to a person talking in various auditory environments, and Figure 1 shows histograms of the results. Among the closed-set questions, this set of questions provided the most insightful results into autistic individuals’ self-perceived listening skills.

Some of the results seem straightforward. Most participants (99%) reported that it was either “very easy” or “fairly manageable” to listen to a person talking in a quiet environment, while situations with background noise were more challenging. Louder environments caused greater difficulty than quieter ones; the proportion of participants selecting “fairly challenging” or “very difficult” was greater for loud mechanical noise (92%) and loud music (96%) than moderate mechanical noise (35%) and moderate-volume music (48%).

However, the complexity of background noise appeared to play a role. At both moderate and loud volumes, music was problematic for a greater proportion of participants than mechanical noise and background voices were even more troubling. For the listening situations with one or two background voices, 88% of participants selected “fairly challenging” or “very difficult,” which is comparable to the results obtained for environments with *loud* mechanical noise or *loud* music. Environments with many other people talking posed the greatest challenges, with 85% of participants indicating that it was “very difficult” to listen to a person talking in such environments. This is greater than the number of people who selected “very difficult” for any other environment, even loud music (81%).

The follow-up questions asked participants to assess whether they had greater listening difficulty than other people their age. For each environment, participants were posed this follow-up question only if they had answered “fairly challenging” or “very difficult” to the previous question. (At the survey design stage, this decision was taken because it would be confusing and inappropriate leading to ask individuals who reported no listening difficulty to subsequently describe the extent of their listening difficulty relative to others.) For all environments, the majority of participants questioned selected “definitely” or “I think so” (see Table 3 and Figure 2). However, the fraction of people selecting “definitely” was substantially higher for environments where one or two people are talking (67%) or many other people are talking (74%), again indicating disproportionate intrusion by background voices.

The results from the questions about the impact of listening difficulties are shown in Table 4 and Figure 3. The majority of participants indicated that listening difficulties had an impact on all aspects of life that we asked about. Most notably, the impact was greatest for participants’ social lives; 91% of participants replied “very much so” or “perhaps” when asked if listening skills affected their social lives. However, more than 90% of participants still selected “very much so” or “perhaps” when asked if listening difficulties affected the impression that they make on other people, and nearly 90% of participants indicated that listening difficulties had stopped them from doing some activities. Additionally, between 70% and 80% of participants replied “very much so” or “perhaps” when asked if listening difficulties affected their education or career or if listening difficulties affected how they felt about themselves.

### Free-text data

Of the 79 evaluable survey participants, 66 supplied free-text data. All six themes in the analysis matrix were represented in these responses, but as expected, the first three themes were most strongly represented. The additional inductive analysis generated 12 new tertiary themes, which were nested under an existing theme in all but one case. A high degree of agreement between the two researchers was reached (97.8% of entries in the matrix were agreed upon). Table 5 lists counts for each theme, subtheme, and tertiary theme (the number of participants whose data were judged as including each concept by one or both researchers). New subthemes (*n* = 2) and tertiary themes (*n* = 12) are highlighted in yellow.

**Table 5.** Responses recorded for each theme.

Subtheme	*n*	Tertiary theme	n
Theme 1: Auditory anomalies (*n* = 57)			
Subtheme 1.1 Difficulty focusing on a voice amid background sounds	22	i. Intense distraction by background voices	13
		ii. Less commonly, distraction by other background sounds (especially irregular, unexpected, and/or high-pitched sounds)	8
		iii. Background sounds need not be loud or numerous	4
Subtheme 1.2 Difficulty distinguishing a voice from background sounds	20	i. Intense difficulty picking out target voice from multiple background voices	11
		ii. Fewer difficulties picking out target voice from nonspeech background noise	10
Subtheme 1.3 Drowning out of a voice by background sounds	23	i. Speech sounds obscured by continuous background sounds (especially loud or low-pitched)	23
		ii. Effect sometimes used beneficially (e.g., using white noise to disguise aversive sounds)	0
Subtheme 1.4 Difficulty orienting to a voice amid background sounds	4	i. Difficulty directing focus to current talker, especially in multitalker environments	2
		ii. Interaction with reliance on visual cues (see Section 3.1)	0
Subtheme 1.5 Loudness discomfort and auditory overload	22	i. Loud and/or complex sound environments causing discomfort/overload	17
		ii. Indirect effects on listening ability, via effects on internal state	7
		iii. Misophonia	6
Subtheme 1.6 Acute hearing sensitivity	8	i. Some advantages (e.g., situational awareness)	3
		ii. Disadvantages (e.g., distraction)	5
		iii. Individual difference (especially for high pitches, lessening with age)	4
Theme 2: Acoustic contributing factors (*n* = 52)			
Subtheme 2.1: Loudness of background sounds	25	i. Loudness can cause difficulties with distinguishing, drowning out, and loudness discomfort	22
		ii. Difficulties with focusing are less loudness-dependent	2
Subtheme 2.2: Diversity of background sounds	43	i. Greater variety of sound sources leads to greater difficulty distinguishing	17
		ii. Room size and reverberation cause difficulties	7
		iii. Number of concurrent talkers (complex and varied effects)	26
		iv. Consistently strong preference for two-person communication	7
		v. Sounds that do not originate from the front	6
Subtheme 2.3: Features of target voice	7	i. Clarity, speed, accent, pitch	7
Subtheme 2.4: Listening to remote audio	16	i. Difficulty listening via phone or video call	9
		ii. Difficulty listening to broadcast or recorded media	8
		iii. Electronic echo	3
Subtheme 2.5: Interference by recorded or broadcast audio	30	i. Interference by background music	25
		ii. Interference greater (or present only) for vocal music	3
		iii. Interference by sound from TV/video	8
Theme 3: Nonacoustic contributing factors (*n* = 43)			
Subtheme 3.1: Visual cues	14	i. Often crucial for orienting to voice	0
		ii. Important for ongoing comprehension (but beware potential negative impact of eye contact)	14
		iii. Can break down in crowded environments	1
Subtheme 3.2: Multisensory processing	22	i. Distraction by other sensory modalities (visual, smell, heat, pain)	20
		ii. Stimulation in preferable multisensory modes can help some concentrate	3
Subtheme 3.3: Cognition and internal state	23	i. Distraction by thoughts and emotions	6
		ii. Motivation, fatigue, and attention levels	12
		iii. Discomfort in crowds	10
Subtheme 3.4: Social cognition and inference to support meaning	2	i. Ability to back fill meaning in utterances using inference and social awareness	2
		ii. A potential area of male-female difference	0
Theme 4: Compounding factors (*n* = 8)			
Subtheme 4.1: Social interaction difficulties	3	i. Listening difficulties are distinct from social difficulties	0
		ii. Listening difficulties and social difficulties have cumulative and interacting effects	2
Subtheme 4.2: Lack of understanding of listening difficulties	6	i. By communication partners	4
		ii. By self	1
		iii. By authority figures (e.g., managers, educators)	1
		iv. By clinicians	2
		v. Inadequacies of existing hearing tests	4
Subtheme 4.3: Concealment of listening difficulties	1	i. Effortful guessing and pretending to keep up	1
		ii. Backfire (missing information, feelings of isolation, anxiety around getting caught out)	0
Theme 5: Impact (*n* = 26)			
Subtheme 5.1: Social participation	12	i. Barrier to participating fully in common social environments	7
		ii. Causing listener to limit duration, frequency, and/or type of socializing	5
		iii. Barrier to relationship building (friends, intimate partners, work colleagues)	0
Subtheme 5.2: Listening effort and listening-related fatigue	8	i. Effort expended on listening leaves fewer mental resources available for comprehension, reflection, and/or retention	5
		ii. Growing fatigue and limited endurance	6
		iii. Increases the cost and diminishes the joy of social participation	0
		iv. “Effort/fatigue cycle”	0
Subtheme 5.3: Emotion	13	i. Negative emotional impact in the moment (e.g., frustration, anxiety, isolation)	11
		ii. Extreme intensity of emotional responses (e.g., distress, nausea, pain)	10
		iii. Persistent impact on emotions and well-being (e.g., loneliness, dread, resentment)	3
Subtheme 5.4: Self-perception	2	i. Self efficacy	2
		ii. Self esteem	0
		iii. Resignation and self-blame	0
Subtheme 5.5: (Perceived) impression made on others	5	i. Inattention/apathy/rudeness/coldness	2
		ii. Incompetence/stupidity	0
Subtheme 5.6: Practical costs	7	i. Time	0
		ii. Money	0
		iii. Occupational/educational attainment	6
Theme 6: Coping mechanisms (*n* = 29)			
Subtheme 6.1: Self awareness and self-advocacy	0	i. Understanding and accepting one's needs	0
		ii. Disclosing hearing problems with & without disclosing autism	0
Subtheme 6.2: Developing auditory skills	3	i. Skills usually self-taught (no standard guidance means unsystematic and uncertain)	3
		ii. Can be supported by positive life challenges	0
		iii. Collaborative development of tactics with Sos	0
Subtheme 6.3: Communication tactics	10	i. Requesting: Getting attention first, clear speech, appropriate positioning, repetition, and clarification	2
		ii. Using visual communication methods (lip reading, observing gestures, signs if appropriate)	7
		iii. Seeking out good/familiar communication partners	0
Subtheme 6.4: Managing the listening environment	9	i. Choosing a preferable listening environment (quiet, calm, small, familiar)	7
		ii. Restricting conversational group size	2
		iii. Requesting reasonable adjustments	1
Subtheme 6.5: Technology	20	i. Improving audio quality: High-fidelity hearing protection, high-fidelity audio equipment	9
		ii. Visual aids to listening: Subtitles, lecture slides	9
		iii. Bypassing adverse listening situations: Online communication methods (e.g. online learning)	0
		iv. Blocking ears	11
Subtheme 6.6: Withdrawal/avoidance	5	i. Limiting time or frequency in adverse listening environments (adaptive)	1
		ii. Building in “down-time” afterward (adaptive)	0
		iii. Maladaptive withdrawal and avoidance (reflexive “snapping” or excessive avoidance)	0
Minor themes			
Unusual parallels between visual and auditory modalities			4

Theme 1 was very strongly represented, with 86% of the free-text sample reporting auditory anomalies. Most of these participants reported listening *difficulties*, despite the free-text questions using neutral language (“easy or difficult,” “harder or easier”). In particular, each of the following was reported as being difficult by ≥20 participants: *Focusing on a voice amid background sounds*, *Distinguishing a voice from background sounds*, *Drowning out of a voice by background sounds*, and *Loudness discomfort/auditory overload*. Some participants reported a combination of these difficulties:

“I don't seem to have the ability to focus on one sound, or block out unwanted sounds. Aside from the difficulty hearing, sound coming from multiple directions is very uncomfortable to me - it puts me into fight or flight mode very easily” [Participant 37]

There was less evidence of *Orienting to a voice amid background sounds* (*n* = 4) and of *Acute hearing sensitivity* (*n* = 8). One fresh concept was *Misophonia* (aversiveness of sounds unrelated to loudness). This was nested under the subtheme *Loudness discomfort and auditory overload* and reported by six participants, often in vivid terms:

“Rustling of paper or plastic bags, snoring, sneezing, my cats licking themselves… noises can drive me to meltdown” [Participant 77]

Theme 2 (*Acoustic factors* influencing ease of listening) was also strongly represented, observed in 79% of the sample. *Loudness of background sounds* (*n* = 25) and *Diversity of background sounds* (*n* = 43) were confirmed as problematic. Within the *Diversity* subtheme, particular emphasis was placed on the difficulty caused by the *Variety of sound sources* (*n* = 17) and the *Number of concurrent talkers* (*n* = 26):

“I've found that the amount of different noises affects me more than the volume of the noises” [Participant 82]

Also placed under this subtheme was a new concept, *Sounds that do not originate from the front*, which was modestly represented (*n *= 6):

“[I] often find sound that is significantly louder in one ear to be more disruptive than were it more evenly balanced between both ears” [Participant 40]

Participants reported a wealth of new concepts relating to broadcast and telecommunications audio, which were not evident in the interview data of Sturrock et al. (2022). Their strong representation in the present survey data prompted the formation of two new subthemes: *Listening to remote audio* (*n* = 16) and *Interference by recorded or broadcast audio* (*n* = 30). The former included *Difficulty listening via phone or video call* (*n* = 9) and *Difficulty listening to recorded or broadcast media* (*n* = 8):

“Processing auditory information on the telephone is difficult for me… I use subtitles when watching tv/movies so I can process what is being said” [Participant 34]

Also under *Listening to remote audio* was *Electronic echo* (*n* = 4), in which participants spoke of difficulties caused by “cross-talk,” “speaker echo,” and “delay in the line”:

“I've had a phone line echo and the echo of my own voice was honestly so distressing it was either disconnect or break down” [Participant 21]

Many participants also reported *Interference by recorded or broadcast audio*, such as *Background music* (*n* = 25) or *TV/video* (*n* = 8):

“I have the hardest time tuning out televisions and talk radio” [Participant 19]

Theme 3 (*Nonacoustic factors* influencing ease of listening) was nearly as strongly represented as Themes 1 and 2, with 43 participants (59%) reporting factors beyond the auditory environment that affect their listening ability. *Multisensory processing* issues (*n* = 22) and the influence of *Cognition and internal state* issues (*n* = 23) were mentioned most frequently. Additionally, a notable fraction of the participants (*n* = 14) mentioned a reliance on *visual cues* and, in particular, the necessity of *Visual cues for ongoing comprehension*:

“I watch people's mouths to help understand” [Participant 48]

A notable number of participants (*n* = 20) reported being *Distracted by other sensory modalities*, as noted in the example below:

“If I have other sensory input happening such as a strong smell or a texture I don't like it makes it very difficult to hear what people are saying.” [Participant 28]

We also introduced a new concept about *Discomfort in crowds* under Theme 3. This does not refer to issues with crowd noise but instead to other sensory issues related to the presence of a large number of people, such as increased physical or visual stimuli from crowds or general anxiety regarding crowded locations, as indicated by this example:

“Being in a social crowded space with lots of stimuli to all of the senses tend to be very difficult.” [Participant 17]

Theme 4 (*Compounding factors*) was hardly represented in participant responses (*n* = 8). Most of these responses fell under the *Lack of understanding of listening difficulties* subtheme (*n* = 6), and half (*n* = 4) of the responses discussed a lack of understanding of listening difficulties *By communication partners*, such as the following response:

“Now I could just ask them to repeat what they said, but the thing is if you are constantly having to ask people that, most people tend to get a bit angry/annoyed I find.” [Participant 8]

We also received four responses discussing the *Inadequacies of existing hearing tests*, which we listed under this theme:

“My hearing has always tested as perfect in quiet environments, but introduce noise and some pitches are worse than others.” [Participant 26]

*Impact* of listening difficulties (Theme 5) was represented in one third (*n* = 26) of responses despite participants not being asked about impacts in any of the free-text questions. Although no single subtheme characterized half or more of the responses under this theme, the *Social participation* (*n* = 12) and *Emotion* (*n* = 13) subthemes were represented more than the others. Many responses within this theme included discussions about *Negative emotional impact in the moment* (*n* = 11) or the *Extreme intensity of emotional responses* (*n* = 10):

“Also little noises that are distracting or annoying, like my cat licking or a light bulb buzzing. They make things hard too, and can even make me so uncomfortable that I vomit.” [Participant 22]

Theme 6 (*Coping mechanisms*) was represented by more than one third (*n* = 29) of the responses. The majority of these responses fall under the *Technology* subtheme (*n* = 20). For example, many participants mentioned earplugs, which we incorporated into a new tertiary theme labeled *Blocking ears*. Importantly, this strategy was used not only to relieve loudness discomfort but also to improve intelligibility:

“I find that wearing earplugs can help drown out a lot of background noise, especially music and traffic noises, and can make it easier to hear someone talking.” [Participant 31]

Finally, we listed as a *Minor theme* four responses that mentioned *Unusual parallels between visual and auditory modalities*. These responses mentioned similarities between an individual's listening difficulties and their issues with processing other sensory stimuli. This quote about sign language was potentially the most enlightening:

“I took a class on American Sign Language at university and there were a couple of times that my brain was unable to process the signs I was seeing that reminded me of when my brain struggles to process auditory information.” [Participant 34]

## Discussion

The most interesting results from the closed-set data were (a) the great difficulty reported by most participants in a variety of relatively common listening environments, (b) more complex background sounds causing greater difficulty, (c) background voices causing exceptional difficulty, (d) the perception by participants that their listening difficulties exceed those of same-aged peers, and (e) the severe impacts of listening difficulties. The fact that many participants find even moderate background noise difficult to handle is important; the fact that 85% find a babble of background voices “very difficult” is even more so, since this typifies many environments that can be essential to our occupational and social lives: cafes, classrooms, parties, offices, restaurants, and so on. There appears to be an association between the complexity of background sounds and their intrusiveness, with music causing greater difficulty than mechanical noise, and voices greater difficulty still. Those participants who report listening difficulties consistently state that their difficulties exceed those of other people their age; “definitely” was the most common answer in every listening situation. Similarly, there is no domain of life (social, occupational, self-conceptual, etc.) that was not said to be “very much” impacted by listening difficulties by most respondents.

**Figure 4.**
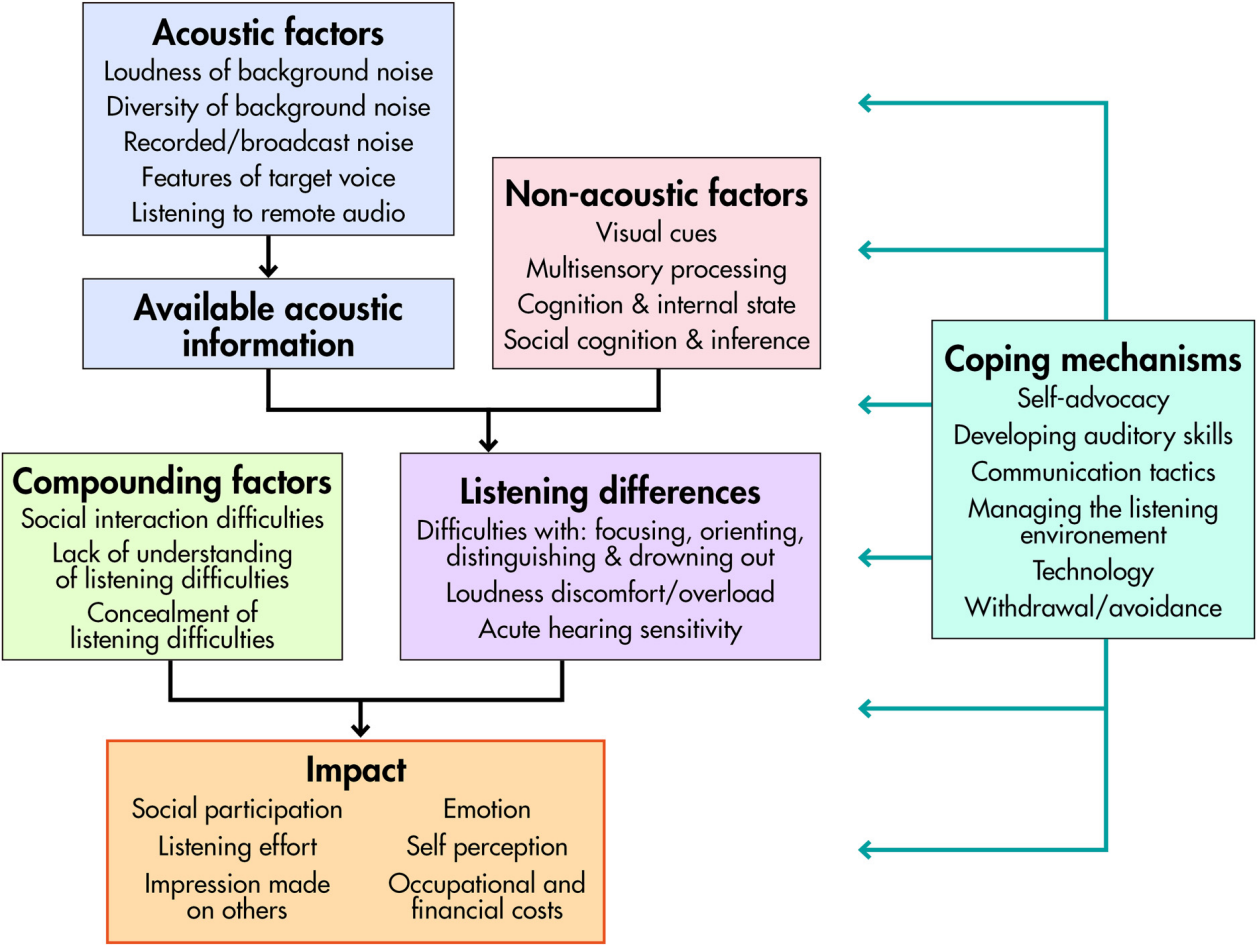
Schematic diagram of the relations between themes.

Meanwhile, the free-text data provide independent insights into autistic speech-perception experiences. Figure 4 illustrates relations among the themes. As expected, content analysis broadly confirmed the first three themes from Sturrock et al. (2022): *Auditory Anomalies* and both *Acoustic* and *Nonacoustic Factors* contributing to listening difficulty. Particularly notable is the diverse array of types of listening difficulty reported, often quite distinct. The fact that these range from “drowning out” of speech by simple noise to distraction by just one or two voices (as well as problems distinguishing voices and auditory overload) suggests that underlying mechanisms may be heterogeneous. This has important implications for laboratory research on speech perception in this population, which may previously have taken insufficient account of heterogeneity. Free-text responses also included many reports of distraction by other sensory modalities and of misophonia, a phenomenon well established in existing autism research (Williams et al., 2021) but less evident in the data of Sturrock et al. (2022). Finally, the free-text data revealed extensive difficulties caused to autistic people by recorded, broadcast, and telecommunications audio that were not previously evident in data gathered by our lab, likely due to our use of oral interviews and resulting self-selection bias. In a post-pandemic world where such technologies are more pervasive than ever, impacts on the autistic community must be considered.

One striking similarity between the free-text and closed-set data is the important role played by noise complexity. The *Acoustic factors* theme is dominated by reports of difficulties caused by diversity in background sounds, music, and concurrent voices. These findings, in combination with our parallel closed-set findings, provide potential clues as to the mechanisms affecting speech perception in some autistic people, again providing important guidance for future research. Commonly used speech-in-noise tasks with simple noise maskers (e.g., white or speech-shaped noise) and lacking real-world cues (such as spatial sound) may be insensitive to the causes of the real-world listening difficulties reported here. Consistent with this reasoning, recent research from the University of Manchester (with 60 autistic people and 56 matched controls) indicates that autistic and nonautistic people perform similarly on a listening task with simple background sounds (speech-shaped noise) but differ on a listening task with complex background noise (spatially separated voices; Blackthorne et al., in preparation). These findings, consistent with our self-report data, support the notion that experimental methods based on detailed autistic insight are more likely to reveal autistic listening differences. More broadly, we contend that future research in this field should be shaped less by researchers’ universal theories of autism and more by insights and feedback from the autistic community.

The capacity of our findings to support valid research into the mechanisms of speech-perception differences is not their only value. These data also support an expanded understanding of the degree and nature of adversity caused to autistic people by listening to difficulties and what might be done to relieve them. In the free-text data, diverse and sometimes severe impacts were widely reported despite the free-text questions not asking about impact. Clearly, autistic people are keen to raise awareness of how critical listening difficulties can be for their daily functioning. Similarly, the free-text data are rich with unprompted reports of coping strategies used to handle listening difficulties, suggesting that the autistic community is keen to share effective tactics. These coping strategies included communication tactics, management of the listening environment, and technological solutions. It would be worthwhile to collate potentially valuable strategies so that they can be shared with the community to aid self-help and perhaps form the basis for clinical guidance.

The listening differences reported in this study tended to be listening difficulties, but it is important to acknowledge that autism may also be associated with positive auditory differences and that our findings are consistent with this view. Remington and Fairnie (2017) argue that autism is associated with generally increased auditory perceptual capacity for attended as well as unattended sounds and that this heightened capacity can represent a skill or a deficit, depending on the task at hand. Consistent with this reasoning and with our data are the qualitative findings of Davies (2022). In accounts of auditory processing experiences posted online by autistic adults, Davies observed three main themes: hyperacusis/auditory overwhelm, difficulty processing a target sound (especially speech) in background noise, and rich processing of soundscapes. Autistic adults reported great pleasure in perceiving complex layers of sound in detail, and it is likely that our study's explicit focus on speech perception made it insensitive to these experiences. Heightened auditory capacity may be troublesome when trying to focus on a target voice in a noisy place but enriching in other circumstances.

Although this questionnaire has yielded substantial new information about autistic individuals’ listening abilities and difficulties, the methods used have some limitations. Firstly, the survey format was likely inaccessible for individuals with substantially limited literacy or intellectual disability. Although questions were posed in relatively simple language, it is likely that responses were issued from a subset of autistic people (those without significant intellectual disability who are comfortable with written communication), limiting generalizability. However, the fact that even highly able participants experience pronounced and varied listening difficulties is telling, and effects on the wider population may be at least as substantial. Secondly, some readers may view our pooling of diagnosed and self-diagnosed autistic people as a limitation. This is not a view we share, given the inequity of access to diagnosis and recent data showing that autistic people exhibit hearing differences regardless of whether they are self-diagnosed or formally diagnosed (Blackthorne et al., in preparation), but we acknowledge this perspective. Similarly, the survey did not verify autistic or nonhearing-impaired status, relying solely on self-report. However, it is worth noting that the upper age limit (55) was selected based on age-related normative hearing data such that substantial presbyacusis should be rare in our sample (Humes, 2020). The study did not incorporate nonautistic individuals, so we cannot know what responses they would have given. As discussed in the Introduction, we do not consider this a study limitation per se, but data from alternative neurotypes may be an interesting target for future research. A further limitation is that the wording of the free-text questions was broad, asking only about easy or difficult listening situations, factors that aid or impair listening, and research priorities. Although this avoided unduly leading participants, it may have been wise to ask also about compounding factors, impacts, and coping strategies, given that the theme list from Sturrock et al. (2022) served as our analysis matrix. Still, the fact that responses on the latter three themes were proffered organically is highly informative, confirming the importance of these concepts to our autistic participants. Finally, our data are purely self-reported, reflecting individuals’ perceptions rather than concrete performance; these should form the basis for subsequent quantitative research.

Based on our integrated findings, we have six recommendations.

1. Autistic insight should inform **better quantitative research** into the mechanisms of listening differences in the autistic population. Self-report data and advice from autistic collaborators should shape the hypotheses and auditory measures, ensuring that listening conditions reflect the aspects of real-world listening that cause difficulty and that data-collection tools are appropriate for autistic as well as neurotypical participants.
2. Future research should take account of **heterogeneity**, disentangling various types of listening differences and potentially exploring how they cluster across the population. Such work will require large sample sizes, so the exploitation of internet-based data collection via computers and smartphones may be fruitful.
3. Methods for the **collection of self-report data** must also be optimized. Restricting data collection to oral communication is clearly problematic, discouraging individuals with listening difficulties and those who find the interpersonal aspects and/or time constraints aversive. Straightforward surveys have their own limitations, neglecting data that could be obtained interactively. An optimal approach may involve innovative compromise, such as the use of nonlive text “interviews” via email, and the choice of interaction methods to suit each individual.
4. There is a pressing need to **raise awareness of autistic listening differences** among various groups, to expand understanding and spur the development of interventions. We suggest that this is true of clinicians, educators, acoustic engineers, communication partners, employers, institutions, policymakers, the wider public, and even self-understanding among some autistic people. Given the substantial difficulties posed by common environments (for example, those with multiple voices, mixed background sounds, and other sensory stimuli), these issues must be understood at an institutional and societal level, not only by the autistic community and supporting professionals (Davies, 2023; Rosas-Pérez et al., 2023).
5. Research should investigate **potentially beneficial interventions** involving all of the above groups with the goal of modifying communication behaviors and listening environments to meet the needs of the autistic community. An effective approach will almost certainly require improved clinical guidance, which our data indicate is lacking. Self-help strategies (such as self-advocacy and communication tactics) and technological solutions (such as high-fidelity ear plugs) will also be useful along with corresponding advice for communication partners. However, in the longer term, we must strive toward wider societal interventions such as improved acoustic standards and guidelines for employers and educators.
6. Effective **coping mechanisms** for handling listening differences could be sought from the community and used as the basis for self-help materials and clinical guidance. These community-generated strategies are likely to include self-advocacy, communication tactics, technological solutions, and means of managing the listening environment.

## Conclusions

Understanding the nature, extent, mechanisms, impacts, and implications of listening differences among the autistic community is an important societal goal. Self-report data from a variety of methods (closed-set, free-text, and previous interview data) now paint a fairly consistent picture. Listening differences are widespread and most often (though not always) consist of listening *difficulties*. Listening difficulties appear to be of several types, ranging from auditory distraction to loudness discomfort, and have substantial impacts on many different areas of daily living. The defining features of the most challenging listening environments (for example, multiple sound sources, background chatter, and multisensory stimuli) are not unusual; they characterize many of our core living and working environments. We must work to better understand the mechanisms underlying listening differences, taking care to disentangle distinct mechanisms and take account of heterogeneity. More immediately, we must raise awareness of autistic listening differences across diverse societal groups and explore a broad range of interventions, not only those wielded by individuals but those applied at institutional and societal levels. In our view, it is time to look critically at the noisy world we have created and to ensure that autistic people are part of the conversation.

## Supplemental Material

sj-docx-1-dli-10.1177_23969415241227074 - Supplemental material for The diversity of speech-perception difficulties among autistic individuals Click here for additional data file. Supplemental material, sj-docx-1-dli-10.1177_23969415241227074 for The diversity of speech-perception difficulties among autistic individuals by George J Bendo, Alexandra Sturrock, Graham Hanks, Christopher J Plack, Emma Gowen and Hannah Guest in Autism & Developmental Language Impairments

sj-docx-2-dli-10.1177_23969415241227074 - Supplemental material for The diversity of speech-perception difficulties among autistic individuals Click here for additional data file. Supplemental material, sj-docx-2-dli-10.1177_23969415241227074 for The diversity of speech-perception difficulties among autistic individuals by George J Bendo, Alexandra Sturrock, Graham Hanks, Christopher J Plack, Emma Gowen and Hannah Guest in Autism & Developmental Language Impairments

sj-xlsx-3-dli-10.1177_23969415241227074 - Supplemental material for The diversity of speech-perception difficulties among autistic individuals Click here for additional data file. Supplemental material, sj-xlsx-3-dli-10.1177_23969415241227074 for The diversity of speech-perception difficulties among autistic individuals by George J Bendo, Alexandra Sturrock, Graham Hanks, Christopher J Plack, Emma Gowen and Hannah Guest in Autism & Developmental Language Impairments

sj-docx-4-dli-10.1177_23969415241227074 - Supplemental material for The diversity of speech-perception difficulties among autistic individuals Click here for additional data file. Supplemental material, sj-docx-4-dli-10.1177_23969415241227074 for The diversity of speech-perception difficulties among autistic individuals by George J Bendo, Alexandra Sturrock, Graham Hanks, Christopher J Plack, Emma Gowen and Hannah Guest in Autism & Developmental Language Impairments
