# Supplementary material for: The diversity of speech-perception difficulties among autistic individuals
Source: Autism Dev Lang Impair. 2024 Jan 27;9:23969415241227074. doi: 10.1177/23969415241227074 (PMC10822079; doi:10.1177/23969415241227074)
Supplement: sj-docx-1-dli-10.1177_23969415241227074 - Supplemental material for The diversity of speech-perception difficulties among autistic individuals [file sj-docx-1-dli-10.1177_23969415241227074.docx]

| **SM1: Consolidated Criteria for Reporting Qualitative Research (COREQ) checklist** | | | |
| --- | --- | --- | --- |
| **Topic** | **Item** | **Guide Questions/Description** | **Reporting location** |
| **Domain 1: Research team and reﬂexivity** | | | |
| ***Personal characteristics*** | | | |
| Interviewer/facilitator | 1 | Which author/s conducted the interview or focus group? | N/A (data collected by survey) |
| Credentials | 2 | What were the researcher’s credentials? E.g. PhD, MD | Paragraph 1 of Methods |
| Occupation | 3 | What was their occupation at the time of the study? | Paragraph 1 of Methods |
| Gender | 4 | Was the researcher male or female? | Paragraph 1 of Methods |
| Experience and training | 5 | What experience or training did the researcher have? | Paragraph 1 of Methods |
| ***Relationship with participants*** | | | |
| Relationship established | 6 | Was a relationship established prior to study commencement? | N/A (data collected by survey) |
| Participant knowledge of  the interviewer | 7 | What did the participants know about the researcher? e.g. personal  goals, reasons for doing the research | N/A (data collected by survey) |
| Interviewer characteristics | 8 | What characteristics were reported about the inter viewer/facilitator?  e.g. Bias, assumptions, reasons and interests in the research topic | N/A (data collected by survey) |
| **Domain 2: Study design** | | | |
| ***Theoretical framework*** | | | |
| Methodological orientation and Theory | 9 | What methodological orientation was stated to underpin the study? e.g. grounded theory, discourse analysis, ethnography, phenomenology,  content analysis | “Analysis of free-text data” section of Methods |
| ***Participant selection*** | | | |
| Sampling | 10 | How were participants selected? e.g. purposive, convenience, consecutive, snowball | “Participants” section of Methods |
| Method of approach | 11 | How were participants approached? e.g. face-to-face, telephone, mail, email | “Participants” section of Methods |
| Sample size | 12 | How many participants were in the study? | “Participants” section of Methods |
| Non-participation | 13 | How many people refused to participate or dropped out? Reasons? | N/A |
| *Setting* | | | |
| Setting of data collection | 14 | Where was the data collected? e.g. home, clinic, workplace | N/A (data collected by survey) |
| Presence of non-  participants | 15 | Was anyone else present besides the participants and researchers? | N/A (data collected by survey) |
| Description of sample | 16 | What are the important characteristics of the sample? e.g. demographic data, date | Table 1 |
| *Data collection* | | | |
| Interview guide | 17 | Were questions, prompts, guides provided by the authors? Was it pilot tested? | N/A (data collected by survey) |
| Repeat interviews | 18 | Were repeat interviews carried out? If yes, how many? | N/A (data collected by survey) |
| Audio/visual recording | 19 | Did the research use audio or visual recording to collect the data? | N/A (data collected by survey) |
| Field notes | 20 | Were ﬁeld notes made during and/or after the interview or focus group? | N/A (data collected by survey) |
| Duration | 21 | What was the duration of the interviews or focus group? | N/A (data collected by survey) |
| Data saturation | 22 | Was data saturation discussed? | N/A (data collected by survey) |
| Transcripts returned | 23 | Were transcripts returned to participants for comment and/or correction | N/A (data collected by survey) |
| **Domain 3: analysis and ﬁndings** | | | |
| *Data analysis* | | | |
| Number of data coders | 24 | How many data coders coded the data? | “Analysis of free-text data” section of Methods |
| Description of the coding tree | 25 | Did authors provide a description of the coding tree? | N/A (coding tree not used) |
| Derivation of themes | 26 | Were themes identiﬁed in advance or derived from the data? | “Analysis of free-text data” section of Methods |
| Software | 27 | What software, if applicable, was used to manage the data? | “Analysis of free-text data” section of Methods |
| Participant checking | 28 | Did participants provide feedback on the ﬁndings? | No |
| *Reporting* | | | |
| Quotations presented | 29 | Were participant quotations presented to illustrate the themes/ﬁndings?  Was each quotation identiﬁed? e.g. participant number | “Free-text data” section of Results |
| Data and ﬁndings consistent | 30 | Was there consistency between the data presented and the ﬁndings? | “Analysis of free-text data” section of Methods |
| Clarity of major themes | 31 | Were major themes clearly presented in the ﬁndings? | “Free-text data” section of Results |
| Clarity of minor themes | 32 | Is there a description of diverse cases or discussion of minor themes? | “Free-text data” section of Results |
