# Supplementary material for: The diversity of speech-perception difficulties among autistic individuals
Source: Autism Dev Lang Impair. 2024 Jan 27;9:23969415241227074. doi: 10.1177/23969415241227074 (PMC10822079; doi:10.1177/23969415241227074)
Supplement: sj-docx-2-dli-10.1177_23969415241227074 - Supplemental material for The diversity of speech-perception difficulties among autistic individuals [file sj-docx-2-dli-10.1177_23969415241227074.docx]

| **SM2: Wording of online questionnaire** | | | |
| --- | --- | --- | --- |
| **Number** | **Question** | | **Possible Responses** |
| 1 | What's your age (in years)? | |  |
| 2 | Are you autistic? | | Diagnosed autistic (including Asperger's)  Seeking a diagnosis  Neurotypical (not autistic) |
| 3 | Have you ever been diagnosed with hearing loss? (By 'hearing loss', I mean abnormal results on a standard hearing test, which involves listening for quiet beeps) | | Yes  No |
| 4 | For each listening situation below, how easy would you find it to hear and understand what's being said? | Listening to a person talking in a quiet environment | Very easy  Fairly manageable  Fairly challenging  Very difficult |
| 5 |  | Listening to a person talking when there's mechanical noise but it's not very loud (e.g. in a vehicle, home appliances) | Very easy  Fairly manageable  Fairly challenging  Very difficult |
| 6 |  | Listening to a person talking when there's mechanical noise and it's very loud (e.g. roadworks, lawnmower) | Very easy  Fairly manageable  Fairly challenging  Very difficult |
| 7 |  | Listening to a person talking when there's music but it's not very loud (e.g. department store) | Very easy  Fairly manageable  Fairly challenging  Very difficult |
| 8 |  | Listening to a person talking when there's music and it's very loud (e.g. concert, nightclub) | Very easy  Fairly manageable  Fairly challenging  Very difficult |
| 9 |  | Listening to a person talking when ONE or TWO other people are talking (e.g. conversations happening nearby, or happening on TV) | Very easy  Fairly manageable  Fairly challenging  Very difficult |
| 10 |  | Listening to a person talking when MANY people are talking (e.g. crowded social gathering) | Very easy  Fairly manageable  Fairly challenging  Very difficult |
| 11 | Do you get the impression that the following listening situations cause you GREATER difficulty than most people your age? | Listening to a person talking in a quiet environment | Definitely  I think so  Probably not  Definitely not |
| 12 |  | Listening to a person talking when there's mechanical noise but it's not very loud (e.g. in a vehicle, home appliances) | Definitely  I think so  Probably not  Definitely not |
| 13 |  | Listening to a person talking when there's mechanical noise and it's very loud (e.g. roadworks, lawnmower) | Definitely  I think so  Probably not  Definitely not |
| 14 |  | Listening to a person talking when there's music but it's not very loud (e.g. department store) | Definitely  I think so  Probably not  Definitely not |
| 15 |  | Listening to a person talking when there's music and it's very loud (e.g. concert, nightclub) | Definitely  I think so  Probably not  Definitely not |
| 16 |  | Listening to a person talking when ONE or TWO other people are talking (e.g. conversations happening nearby, or happening on TV) | Definitely  I think so  Probably not  Definitely not |
| 17 |  | Listening to a person talking when MANY people are talking (e.g. crowded social gathering) | Definitely  I think so  Probably not  Definitely not |
| 18 | Are there any listening situations you find particularly easy or difficult? | | [Free-text] |
| 19 | Is there anything that makes listening harder or easier? (E.g. location, how many people are there, type of background noise, how loud it is, other sensory stimuli, etc.) | | [Free-text] |
| 20 | Have listening difficulties ever... | ... stopped you from doing anything? | Very much so  Perhaps  Probably not  Definitely not |
| 21 |  | ... affected your education or career? | Very much so  Perhaps  Probably not  Definitely not |
| 22 |  | ... affected your social life? | Very much so  Perhaps  Probably not  Definitely not |
| 23 |  | ... affected how you feel about yourself? | Very much so  Perhaps  Probably not  Definitely not |
| 24 |  | ... affected the impression you make on other people? | Very much so  Perhaps  Probably not  Definitely not |
| 25 | Do you think that research on speech perception in autistic people is a good idea? | | Yes  No |
| 26 | Is there anything you think this research should look into (especially if it hasn't been mentioned above)? | | [Free-text] |
