# Supplementary material for: The diversity of speech-perception difficulties among autistic individuals
Source: Autism Dev Lang Impair. 2024 Jan 27;9:23969415241227074. doi: 10.1177/23969415241227074 (PMC10822079; doi:10.1177/23969415241227074)
Supplement: sj-docx-4-dli-10.1177_23969415241227074 - Supplemental material for The diversity of speech-perception difficulties among autistic individuals [file sj-docx-4-dli-10.1177_23969415241227074.docx]

| **SM4: Representative quotes for new themes** | | | |
| --- | --- | --- | --- |
| **Theme** | **Subtheme** | **Tertiary Theme** | **Representative quote** |
| Theme 1: Auditory anomalies | Subtheme 1.5 Loudness discomfort and auditory overload | iii. Misophonia | “Also little noises that are distracting or annoying, like my cat licking or a light bulb buzzing. They make things hard too, and can even make me so uncomfortable that I vomit.” [Participant 22] |
| Theme 2: Contributing factors (acoustic) | Subtheme 2.2: Diversity of background sounds | v. Sounds that do not originate from the front | “I know I have particular difficulty with differences in what I'm hearing between left and right ear. I find wearing only one headphone/earbud disorienting for example and often find sound that is significantly louder in one ear to be more disruptive than were it more evenly balanced between both ears.” [Participant 40] |
|  | Subtheme 2.4: Listening to remote audio | i. Difficulty listening via phone or video call | “Phone/video calls - if you're speaking to me on the phone and there is any kind of noise going on in the room that I am in (music, people talking, lawnmowers etc.) I will really struggle to hear you.” [Participant 27] |
|  |  | ii. Difficulty listening to broadcast or recorded media | “Dialogue in movies are very difficult, I cannot filter the sound effects, I always use subtitles.” [Participant 32] |
|  |  | iii. Electronic echo | “A few times I've had a phone line echo and the echo of my own voice was honestly so distressing it was either disconnect or break down.” [Participant 21] |
|  | Subtheme 2.5: Interference by recorded or broadcast audio | i. Interference by background music | “Music at anything above a very low background erases my ability to understand what people say.” [Participant 25] |
|  |  | ii. Interference greater (or present only) for vocal music | “I find it more challenging to hear what somebody is saying if the background noise contains voices. I don't mind instrumentals playing in the background on a low volume but sometimes at work when the radio is on and there's someone singling it can be hard to follow a conversation because I can't tune out different sounds very well.” [Participant 27] |
|  |  | iii. Interference by sound from TV/video | “I have the hardest time tuning out televisions and talk radio.” [Participant 19] |
| Theme 3: Contributing factors (non-acoustic) | Subtheme 3.3: Cognition and internal state | iii. Discomfort in crowds | “Being in a social crowded space with lots of stimuli to all of the senses tend to be very difficult.” [Participant 17] |
| Theme 4: Compounding factors | Subtheme 4.2: Lack of understanding of listening difficulties | v. Inadequacies of existing hearing tests | “My hearing has always tested as perfect in quiet environments, but introduce noise and some pitches are worse than others.” [Participant 26] |
| Theme 6: Coping mechanisms | Subtheme 6.5: Technology | iv. Blocking ears | “I find that wearing earplugs can help drown out a lot of background noise, especially music and traffic noises, and can make it easier to hear someone talking.” [Participant 31] |
| Miscellaneous | | i. Unusual parallels between visual and auditory modalities | “I took a class on American Sign Language at university and there were a couple of times that my brain was unable to process the signs I was seeing that reminded me of when my brain struggles to process auditory information.” [Participant 34] |
